# Comparison of Growth and Chemical Profile of Diatom *Skeletonema grevillei* in Bioreactor and Incubation-Shaking Cabinet in Two Growth Phases

**DOI:** 10.3390/md20110697

**Published:** 2022-11-07

**Authors:** Roberta Frleta, Marijana Popović, Tvrtko Smital, Vida Šimat

**Affiliations:** 1 Center of Excellence for Science and Technology-Integration of Mediterranean Region (STIM), Faculty of Science, Ruđera Boškovića 35, University of Split, 21000 Split, Croatia; 2 Department of Applied Science, Institute for Adriatic Crops and Karst Reclamation, Put Duilova 11, 21000 Split, Croatia; 3 Division for Marine and Environmental Research, Ruđer Bošković Institute, Bijenička 54, 10002 Zagreb, Croatia; 4 University Department of Marine Studies, University of Split, Ruđera Boškovića 37, 21000 Split, Croatia

**Keywords:** diatom, bioreactor, incubation-shaking cabinet, derivatized compounds, antioxidant activity

## Abstract

Marine microalgae, diatoms, are considered a source of a wide range of high-value compounds, and numerous studies indicate their biotechnological potential in the food and feed industry, cosmetic industry, nanotechnology, pharmaceutical industry, biodiesel production, fertilizers, and wastewater treatment. The aim of this study was to compare the growth, chemical profiles, and antioxidant activity of the diatom *Skeletonema grevillei* cultivated in a bioreactor and an incubation-shaking cabinet at different growth phases (after 192 and 312 h). Growth was monitored by evaluating cell density with the Sedgewick Rafter chamber, and the collected biomass was extracted with 70% ethanol assisted by ultrasound. Extracts were evaporated to dryness and compounds were identified in derivatized form by gas chromatography and mass spectrometry (GC-MS) analysis, while antioxidant capacity was evaluated by DPPH and ORAC. Significantly faster growth was observed in the bioreactor than in the incubation-shaking cabinet. Oleamide, palmitelaidic acid, glycerol monostearate, myristic acid, cholesterol, eicosapentaenoic acid, 1-monopalmitin, and 24-methylene cholesterol were identified as the major compounds in both systems. Among them, oleamide was the dominant compound in both systems. It is also shown that prolonging the cultivation period had a direct effect on increasing the extract yield. The highest DPPH inhibition (11.4 ± 1%) and ORAC values (93.3 ± 8.4 mM TE) were obtained for the *S. grevillei* extract recovered from the bioreactor after 312 h. The obtained results contribute to the possibility of using *S. grevillei* for various biotechnological applications in the future.

## 1. Introduction

Market demand is changing rapidly, directing research toward finding new ways to use marine resources as a renewable and sustainable source of compounds that can be used in a variety of industries. Among the great biodiversity found in the oceans and seas, marine microalgae are recognized as microorganisms with exceptional biotechnological potential, especially in industries directed to production of pharmaceuticals, functional food ingredients, and food products [1]. Although the application of microalgae is mainly related to the food industry, many species have potential for wastewater treatment, biodiesel production, and nutraceuticals [2]. Despite their great potential, the wider application of a larger number of species is still not economically viable due to numerous challenges, mainly related to technological barriers.

Diatoms are photosynthesizing microalgae with cell walls of transparent opaline silica containing compounds such as pigments, sterols, and fatty acids [3]. Currently, the most commercially important diatom species is *Dunaliella salina*. As the biotechnological potential of diatoms is constantly being discovered, the number of studies on different species is increasing. Diatoms have shown promising features that make them ideal candidates for this form of production, as their intracellular content can be used for the production of biodiesel and valuable food components, and the silicate shell can be used as a material for nanotechnology [4]. In terms of commercial use, the genus *Skeletonema* is an unconventional group of microalgae, but since it is non-toxic, it is suitable for use in the food industry [5]. In fact, the Food and Drug Administration considers *Skeletonema* sp. as generally recognized as safe (GRAS) [6]. Some species such as *S. marinoii* have been described in the literature [7,8]; however, there are no reports on *S. grevillei*. Nevertheless, the cultivation of microalgae for wider use slowed down due to technological obstacles, mainly related to the optimization of growth, harvesting, and extraction efficiency of targeted compounds [9]. In addition to the removing of technological barriers, cultivation should be directed toward the utilization of all production components (bio-based refinery), which would increase the economic profitability and environmental sustainability of the commercial use of diatoms.

Diatoms are among the most flexible and productive eukaryotic microalgae characterized by exceptional adaptability to changing environmental conditions (e.g., temperature and light). Mechanisms such as extracellular polysaccharide layers, cell wall thickness, and morphologically distinct resting stages minimize the potential damage caused by stress conditions [10,11,12]. On the other hand, their abundance in cultivation under laboratory conditions is much lower than in the natural environment. Therefore, optimization of cultivation and selection of suitable initiators for higher production of metabolites is of utmost importance. Increased production of primary and secondary metabolites is associated with stressful cultivation conditions (e.g., nutrient deficiency, non-optimal temperature, different light sources), while chemical characterization of the intracellular content (primary and secondary metabolites) is quite complex. Promoting metabolite production through nutrient deprivation and introducing other stress growth conditions often result in lower biomass yield, which is a common problem in microalgae production. The main challenges are obtaining a sufficient amount of biomass for biotechnological tests, problems related to the effects of nutrients and environmental changes on biosynthesis, and impurities [4]. Conventional cultivation in incubators typically produces little biomass, and the cost of the production effort exceeds the biomass yield and profitability. In 1997, Fukami et al. [13] developed the first bioreactor to promote biomass production of the diatom *Nitzschia* sp. In general, cultivation in bioreactors achieves greater homogeneity so that all cells have the same availability of key factors for successful growth, such as nutrients and light. While the production of higher amounts of primary metabolites is associated with optimal growth conditions, the accumulation of secondary metabolites is usually associated with stressful conditions [14]. Microalgae are known to produce high-value bioactive compounds such as polyunsaturated fatty acids (PUFA), carotenoids, phenolic compounds, sterols, and sulfated polysaccharides with many health and therapeutic benefits [6,15]. Nevertheless, there is little information on the cultivation potential and chemical profile of the genus *Skeletonema*. Some species such as *S. marinoii*, *S. dohrnii* and *S. costatum* have been previously studied [7,16,17,18]; however, there are no reports on *S. gravileii*. In a nutrient-deprived environment, *Skeletonema* species are able to increase lipid production [10], and even produce higher amounts of omega-3 eicosapentaenoic acid (EPA) [19]. Under light and temperature stress, these microorganisms produce components that exhibit antioxidant activity, while the selection of a suitable cultivation system can significantly increase biomass yields, i.e., extract yields [20,21]. In addition, metabolic processes in diatoms are extremely fast, and the production of various molecules may also depend on the growth phases [8].

Biotechnological improvements are possible by selecting suitable microalgal strains capable of producing large amounts of various bioactive compounds under optimal conditions. Such a form of microalgae cultivation would make commercialization faster and more profitable. Therefore, the aim of this study was to determine and compare the growth of *S. grevillei* in a standard F/2 medium using two cultivation systems (bioreactor and incubation-shaking cabinet). In addition, ethanolic extracts were prepared for each system at two growth stages and their chemical profiles (dominant compounds) and antioxidant potential were determined.

## 2. Results and Discussion

### 2.1. Comparison of Growth Curves in BRC and EIS

Considering that extending the duration of the cultivation period beyond the stationary phase would result in the decay of diatom cells, the end of cultivation in this study was defined as the time at which one of the systems reached maximum growth. This was considered the beginning of the stationary phase and in the bioreactor system (BRC) it was reached after 312 h.

**Figure 1:**
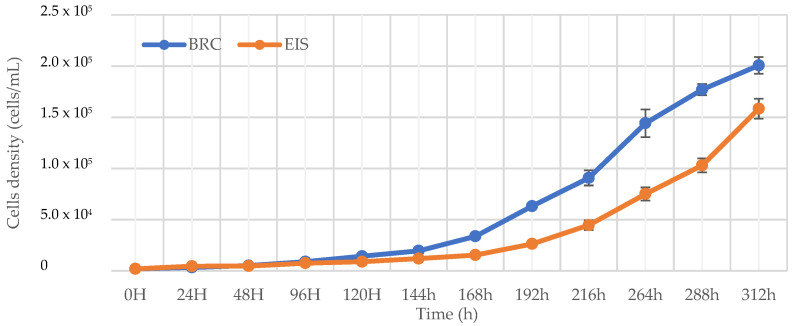
Growth curves for *Skeletonema grevillei* in a bioreactor and incubation-shaking cabinet during cultivation period of 312 h.

**Figure 2:**
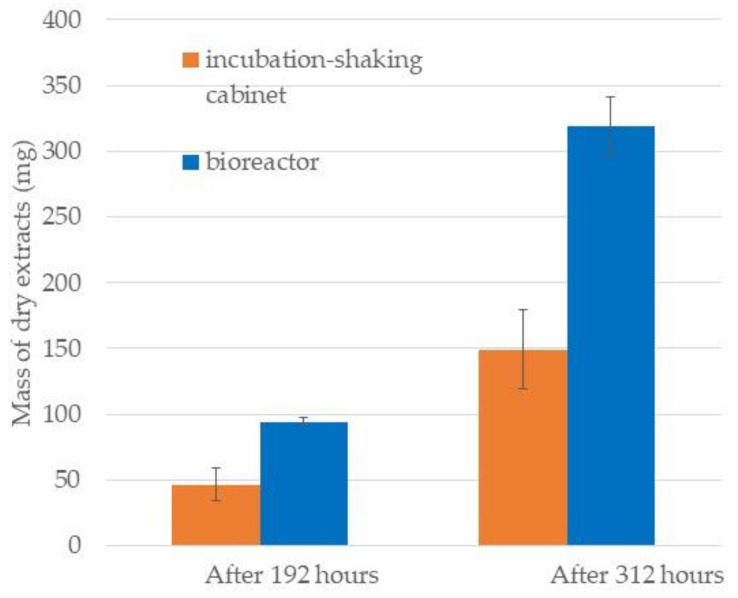
The average mass of dry *Skeletonema grevillei* extracts from bioreactor and incubation-shaking cabinet obtained from total diatom biomass after cultivation periods of 192 and 312 h.

The results of growth in a BRC and the incubation-shaking cabinet (EIS) system during the cultivation period are shown in Figure 1. First, the growth rate in each system over time was examined. After 144 h of cultivation, a statistically significant (*p* < 0.05) change (onset of exponential growth) was observed in the EIS system, while in the BRC system this was observed one day earlier. During the first 144 h, there was no statistically significant difference (*p* > 0.05) in cell number between the EIS and BRC systems. A statistically significant *(p* < 0.05) difference between the systems was first observed after 168 h and persisted until the end of cultivation. The total mass of dry extracts collected in the exponential and at the beginning of the stationary growth phase is shown in Figure 2. The weight of the dry extract was higher in the BRC system at both collection times. The results indicate that the higher homogeneity in the BRC system provided by the impeller and airflow was crucial for the uniform availability of light and nutrients, which consequently led to faster growth of diatoms in this system.

Since the early days of diatom cultivation in bioreactors, it has been known that cell homogeneity allows maximum utilization of limiting nutrients [13]. Although the lack of nutrients often stimulates the metabolism to produce secondary metabolites, these conditions cause disturbances in the growth of diatom cultures. Deficiencies in nutrients such as N and Si negatively affect growth, motility, and often cell morphology, but they are found to be important in stimulating diatom metabolism to produce more valuable metabolites [22,23,24,25]. Ramirez et al. [26] compared the growth of two diatoms, *N. epithemioides* and *Nitzschia* sp. at different temperatures, light intensities and silicate levels. The addition of silicate was found to be more suitable to stimulate growth than modification of light and temperature. Raniello et al. [20] recorded greater biomass production of the diatom *Cocconeis neothumensis* under different light and nutrient conditions in a bioreactor than in a batch culture in Petri dishes. Significantly higher extract yield (24 ± 5 mg of dry extract) was obtained in the bioreactor compared to batch cultures (17 ± 3 mg of dry extract), which is comparable to the results of extract yield between BRC and EIS systems in this study [20].

### 2.2. Identification of Compounds by GC-MS

**Figure 3:**
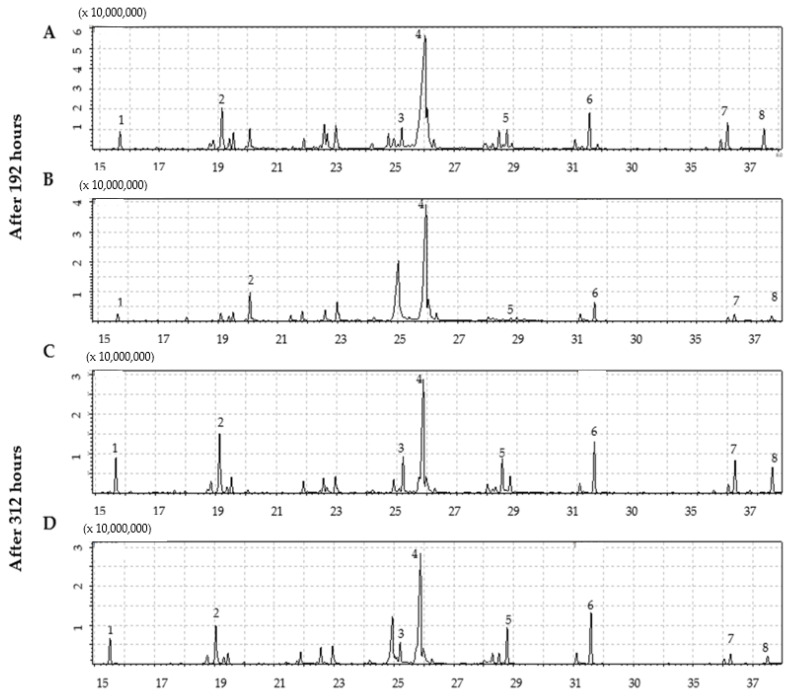
The chromatograms of derivatized dominant compounds in *Skeletonema grevillei* extracts tentatively identified by GC-MS in: (**A**,**C**) bioreactor, (**B**,**D**) incubation-shaking cabinet (**1**—myristic acid, **2**—palmitelaidic acid, **3**—eicosapentaeonic acid, **4**—oleamide or 9- octadecenamide, **5**—1-monopalmitin, **6**—glycerol monostearate, **7**—cholesterol, **8**—24-methylene cholesterol).

The compounds identified in a BRC, and EIS are listed in Table 1 and Table 2 along with their retention index, percentage of similarity, proportion, and molecular weight. The retention times and MS *m/z* of the compounds is given in the Supplementary Material (Table S1). The chromatograms obtained are shown in Figure 3. Since the method is not quantitative, the proportions of the compounds refer only to the injected sample. The amount of each compound in the injected sample volume is expressed as a percentage calculated based on the areas obtained. In Table 1 and Table 2, for the compounds identified in derivatized or non-derivatized form, only those with ≥85% similarity spectra and matching RI were considered. In addition, all compounds with similarity less than 90% were confirmed in non-derivatized form. All listed compounds were confirmed according to previous studies on the chemical profiling of disatoms [7,27,28].

The difference in the number of compounds identified in the BRC system after 192 and 312 h of cultivation was 44 and 42 compounds, respectively. Compared to BRC, a lower number of compounds were identified in both phases of the EIS system, 33 after 192 h and 34 after 312 h of cultivation. Oleamide (also known as 9-octadecenamide or (*Z*)-9-octadecenamide) was dominant in both cultivation systems and at both phases. After 192 h, the percentage of oleamide in BRC and EIS was 49.84% and 73.31%, respectively. In both systems, there was a significant decrease with the extension of the cultivation period, by 16.19% in BRC and 37.78% in EIS.

Compounds with a proportion higher than 5% were palmitelaidic acid, glycerol monostearate, cholesterol, 24-methylenecholesterol, myristic acid, and EPA acid in BRC and glycerol monostearate, palmitelaidic acid, myristic acid, and 1-monopalmitin in EIS. The second most abundant compound in BRC was palmitelaidic acid, while in EIS it was glycerol monostearate. Between the two samplings, the proportion of palmitelaidic acid increased from 7.37% to 12.15% in BRC and from 1.63% to 7.89% in EIS. On the other hand, the proportion of glycerol monostearate increased 3-fold in EIS (from 3.52% to 10.64%) while the increase was lower in BRC at the end of the cultivation period (from 5.92% to 9.30%). To our knowledge, this is the first time it has been identified in marine diatoms. In both systems, myristic acid increased by 3.95% in EIS and 3.53% in BRC, with an additional culture period of 120 h. After 312 h of cultivation, EPA was identified in both systems but the proportion in BRC was higher than in EIS, 6.40% and 3.78%, respectively. In addition, after 192 h, EPA was not detected in the EIS, while in BRC, it was recorded in proportion of 3.29%. The proportion of 1-monopalmitin in BRC remained almost unchanged (from 2.91% to 2.89%), and the same compound showed a sharp increase in EIS (from 0.47% to 6.92%) with the extension of cultivation.

In addition, an increase in tridecanoic acid, loliolide, pentadecanoic acid, palmitic acid, phytol, 2-monostearin, and loliolide was observed in both systems over cultivation time, while the proportion of decanoic acid remained unchanged. At the end of the cultivation period, some newly identified compounds in a proportion of less than 1%, were malic acid, (Z)-3-hexenyl-β-glucopyranoside, palmitoleamide, 1-octadecanol, desmosterol in BRC and butanedioic acid, hexadecanenitrile, 1-hexadecanol in EIS.

There were eight compounds that were predominant in both systems. These are listed in Table 3, with chemical formula and structure and biological activity reported in recent studies.

Oleamide is an amide derived from oleic acid. It is found in green algae such as *Chromochloris zofingiensis* and in few terrestrial plants. There are numerous biological activities related to this compound [29,30,31]. In general, oleamide from green algae is used in the treatment/prevention of arthritis, atherosclerosis, thrombosis, and cancer [32]. It was reported that oleamide from terrestrial plants (*Ipomoea* and *Dillenia* species) excretes anti-inflammatory properties [29]. Furthermore, oleamide from the fungus *Colletotrichum gloeosporioides* has confirmed antimicrobial activity against *Staphylococcus aureus* (zone of inhibition = 25 mm) [33]. The extract of *Diaporthe schini*, in which oleamide was a dominant metabolite, also showed antimicrobial activity against *S. aureus* (MIC = 125 uL/mL) [34]. Furthermore, antifungal, and antimicrobial activity of oleamide from cinnamon bark was recorded against *Aspergillus flavus* and *Klebsiella pneumonia* [35]. It is also recognized as a potential algicide in the control of cyanobacterial blooms, especially against *Microcystis aeruginosa* NIES -843, a toxin-producing cyanobacterium [30]. Oleamide was found to be the main metabolite among the various bioactive compounds found in the green microalgae *Tetraselmis tetrathela* when cultivated at a salinity of 30 ppm [36].

Fatty acids are known for their antimicrobial and antibiofilm activity. Monounsaturated palmitelaidic acid showed antibiofilm activity against Escherichia coli, inhibiting 25% of biofilm formation at 256 µg/mL, while inhibition of S. aureus was 21% at a concentration of 16 µg/mL [37]. In general, diatoms are known producers of long-chain fatty acids such as omega-3 EPA [38]. EPA is known for its health-beneficial effects, so it is used in dietary supplements and nutraceuticals. During the stationary growth phase of diatoms, the limited amount of nutrients from the culturing media is promoting the production of EPA in the cells [39]. In our study, we reported a higher accumulation of EPA in a BRC during the stationary growth phase, which was likely caused by nutrient deficiency. Moreover, myristic acid was identified as the most abundant fatty acid in *S. grevillei* for both cultivation systems. Marzec et al. [27] also found the myristic acid to be the most abundant fatty acid in the diatom *Halamphora* cf. *salinicola* (strain SZCZM1454A). Myristic acid has been described for a variety of biological activities, including antifungal, antiviral, anticancer, and antiparasitic activities [40].

An important cellular metabolite in triacylglycerol (TAG) biosynthesis is 1-monopalmitin. When TAGs are synthesized more intensively, a decrease in the content of 1-monopalmitin can be observed [49]. In *S. grevillei* we observed the decrease of 1-monopalmitin, probably due to the synthesis of fatty acids such as eicosaonic, EPA, and 5,8,11-eicosatrienoic acids.

Glycerol monostearate is known as the glycerol ester of stearic acid. There is no available literature on this compound from diatoms. So far it has been extracted from plant *Dichapetalum filicaule* and reported for antihelminthic activity against hookworms [41]. In general, glycerol monostearate from terrestrial plants is widely used to extend shelf-life and as an emulsifier in the food industry.

**Figure 4:**
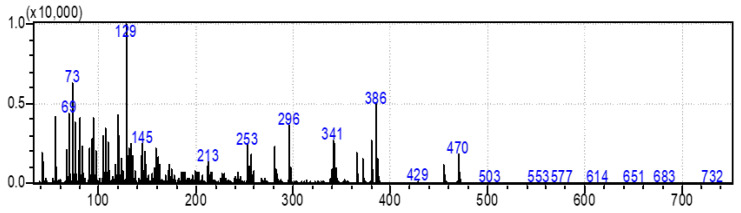
Mass spectra of derivatized sterol compound 24-methylene cholesterol in *Skeletonema grevillei* extracts.

One of the most interesting pieces of information about the metabolic system of diatoms is their ability to synthesize animal and plant sterols [15]. In this study, the results showed a three-fold higher production of sterols in the BRC (12.70%) system compared to the EIS (3.69%) at the end of the cultivation period. The same ratios in sterol production between BRC and EIS can also be seen after 192 h of cultivation. The most abundant animal sterol in diatoms is cholesterol, which has a wide range of biological activities [42,50]. We identified three sterol compounds in derivatized extracts of *S. grevillea,* cholesterol, desmosterol, and isofucosterol. In addition to cholesterol, 24-methylene cholesterol was found among the dominant compounds. The proportion of 24-methylene cholesterol in BRC was 3.44% higher than in EIS at the end of the cultivation period. The mass spectra of the fourth sterol compound, 24-methylenecholesterol in derivatized form (Figure 4) was identified at a retention index of 3259. Since it was not available from the commercial libraries, the compound was confirmed in its non-derivatized form and in alignment with the literature data. Yang et al. [28] have provided the GC-MS spectrum for BSTFA derivatized 24-methylene cholesterol, which is in accordance with our results, while Brooks et al. [51] provided RI for the BSTFA derivatized 24-methylene cholesterol, which is also in alignment with our data.

A wide range of biological activities such as anticancer, anticardiac, anti-inflammatory, antimicrobial, anti-psychotic, and antioxidative have been reported for cholesterol [42]. In addition, Cutignano et al. [48] reported the anticancer activity of 24-methylene cholesterol against breast and lung cancer.

These results are consistent with previous studies that characterized the genus *Skeletonema* by a high content of sterols. Sterols with chemotypes of animals (cholesterol and desmosterol), plants (24-methylene cholesterol) and alga (fucosterol) were previously identified in the diatom species *S. marinoi* [7]. Cultivation conditions, especially a decrease in the amount of available nutrients, cause stress to microalgal cells that stimulates the metabolic production of sterols [52]. The modification of the cultivation conditions significantly affects the sterol profile [15]. A similar impact was observed in this study, at the beginning of the stationary phase in BRC when the amount of nutrients was limited higher sterol content was observed. Based on the sterol profile, it was originally hypothesized that diatoms could synthesize sterols by cyclization of lanosterol and cycloartneoids [7,50,53]. However, Gallo et al. [7] confirmed that the *S. marinoi* strain does not possess genes for lanosterol synthetase, but that desmosterol and cholesterol are produced via cycloartenoid pathways [15]. Since *S. marinoi* belongs to the same order as *S. grevillei*, it can be hypothesized that the biosynthesis of sterols occurs in a similar manner.

### 2.3. Antioxidation Activity Assays

**Figure 5:**
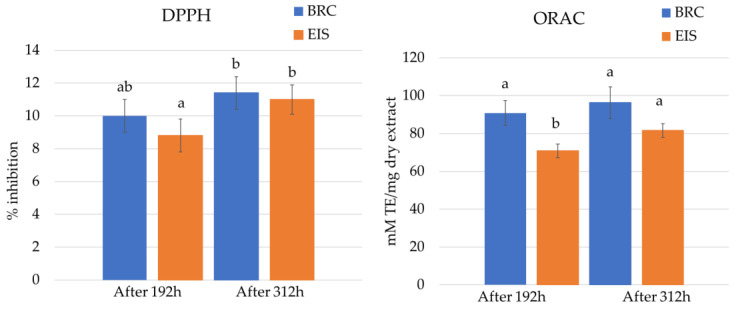
DPPH inhibition (**left**) and ORAC (**right**) results of *Skeletonema grevillei* extracts from bioreactor and incubation-shaking cabinet after 192 and 312 h. Different letters denote statistical difference (*p* < 0.05).

The results of antioxidant activity (DPPH and ORAC) of diatom extracts are shown in Figure 5. At the tested concentration (10 mg dry extract/mL), the DPPH radical inhibition of the extracts from both systems was low. The highest DPPH inhibition (11.4 ± 1%) was observed for the extract from BRC after 312 h. Extracts from the stationary phase showed higher inhibition results for both systems.

In addition to the DPPH method, the ORAC method was chosen because it best simulates in vivo conditions and provides results for antioxidant levels equivalent to those in biological systems [54]. For the ORAC assay, the diatom extracts were diluted 10-fold. The highest ORAC value of 93.3 ± 8.4 mM TE was obtained for the extract from BRC after 312 h. Similar to DPPH inhibition results, ORAC values increased over cultivation period.

Fatty acids and sterols are recognized as compounds with strong antioxidant activity [42,55]. The increase in EPA and cholesterol over the cultivation period might be the reason why the antioxidant activity of *S. grevillei* extracts also increased. A statistically significant difference (*p* < 0.05) in antioxidant activity was detected between cultivation systems at the same growth stage for ORAC, but not for DPPH. 

Antioxidant activity of aqueous and ethanolic extracts of marine microalgae *Tetraselmis* sp. IMP3, *Tetraselmis* sp. CTP4, and *Skeletonema* sp. was evaluated using the methods ferric ion reducing antioxidant power (FRAP), DPPH, and 2,2′-azino-bis (3-ethylbenzothiazoline-6-sulphonic acid) (ABTS) [56]. The highest antioxidant activity was found with the ABTS method, reaching a reduction of more than 80% in aqueous extracts of *Skeletonema* sp. while the results of ethanolic extracts obtained with the DPPH method were comparable to those obtained in the present study.

## 3. Materials and Methods

### 3.1. Chemicals

LiCrosolv Ethanol (gradient grade), Sigma Aldrich (St. Louis, MO, USA) was used for extraction. In GC/MS analysis NO-Bis(trimethylsilyl)trifluoroacetamide (BSTFA) from Sigma-Aldrich (St. Louis, USA) was used as a derivatization reagent while dichlormethane (Gram-mol, Croatia) was used as a solvent for the non-derivatized sample.

### 3.2. Experimental Design

The strain of *S. grevillei* (CIM876), isolated from the northern Adriatic Sea, was donated from the culture collection of the Center for Marine Research of the Ruđer Bošković Institute (Rovinj, Croatia). The diatom was cultured in F/2 medium in two parallel systems: (i) in a bioreactor (BRC) (BiostatB Twin, Sartorius, Goettingen, Germany), (ii) in a 5-L Erlenmeyer flask set in an incubation-shaking cabinet (EIS) (Certomar T plus, Sartorius, Goettingen, Germany). Two replicas were used for each system, each containing 2 L of F/2 medium inoculated with 50 mL of *S. gravillei* (9 × 10^4^ cells/mL). The medium was prepared according to the previously described recipe [57]. 

In both systems, diatoms were cultured at 18 °C with a light–dark photoperiod set to 12 h. The light intensity was 7500 lux in both cultivation systems. In BRC, a LED light (Led GNC Minu Deep AM140, Sicce, Pozzoleone, Italy) was used (a combination of cool white and blue), and fluorescent lamps (Fluora T8, Osram, Garching, Germany) were used in EIS. In BRC, the diatoms were stirred with an impeller at 70 rpm and the air flow rate set at 1 L/min, while in EIS they were shaken at a low speed (50 rpm) to avoid spillage.

### 3.3. The Growth Curve Determination

To compare the growth rate in BRC and EIS systems, 5 mL of the cultures were collected every 24 h of the cultivation period under sterile conditions. The growth curve was determined by counting the cells in a Sedgewick Rafter chamber using a light microscope (Sundew MCXI600, Micros, St. Veit/Glan, Austria) at 200xX magnification [58]. 

### 3.4. Collection and Extraction of Diatom Biomass

The biomass was collected at the exponential and at the beginning of stationary growth phases. These were determined at 192 and 312 h of culturing. Using the filtration glass microfiber filters (Grade GF/F Whatman) at 3.12 psi pressure the biomass was collected and transferred to falcon tubes and re-suspended in 10 mL of 70% ethanol. Extraction was performed in duplicate by ultrasound-assisted extraction (UAE) using an ultrasound probe (Sonoplus HD 3200, Bandalin, Berlin, Germany) set at 20 kHz ± 500 Hz. The extraction was performed for 5 min, at an amplitude of 30% and treatments of 20 s. The break between treatments was 40 s. The samples were centrifuged (Lynx 4000 centrifuge, Thermo Scientific, Waltham, MA, USA) at 29,097× *g*, at 4 °C for 30 min to separate the silicate frustule. The collected supernatant was filtered through a sterile CA filter with a pore diameter of 0.2 µm (LGG, Meckenheim, Germany) and evaporated in a centrifugal evaporator (RC10-22, Jouan, Herblain, France) until completely dry. The mass of the dry extract was recorded.

### 3.5. Identification of Compounds by GC/MS

The identification of compounds was done according to Torras-Claveria et al. with minor modifications [59]. Briefly, derivatizing agent (BSTFA) (50 μL) was added to the dry extracts. The identification of the compounds was determined using gas chromatography (GC, Nexis GC-2030, Shimadzu, Kyoto, Japan), coupled with a mass spectrometry (MS) detector (Shimadzu QP2020 NX), equipped with a split/splitless injection port. The analyses were performed using a fused silica capillary column (length 30 m × inside diameter 0.25 mm i.d., film thickness 0.25 m) (SH-5MS, Shimazu, Japan). Ultra-pure helium was used as carrier gas with a 1 mL/min flow rate. Analyses were performed with MS full scan (35–750 m/z). A perfluorotributylamine was used for calibration of the mass spectrometer, at an electron impact ionization energy of 70 eV. The column temperature program was oven equilibration time of 3 min; initial temperature 120 °C for 3 min, increased to 292 °C at a rate of 5 °C /min, then increased to 320 °C at a rate of 30 °C/min and held isothermal for 17 min. Identification of the compounds in derivatized extracts was performed by comparing their trimethylsilyl (TMS) derivative mass spectra and GC retention times with Wiley 12 & NIST 2020 databases [60,61]. All samples were injected and analyzed in duplicate.

Since commercial libraries did not offer adequate mass spectra for the identification of compounds in derivatized forms, the same samples were analyzed in non-derivatized form as well. The analyses were performed according to the same protocol, but 50 μL dichloromethane was used instead of the derivatizing agent. The derivatized and non-derivatized compounds were identified by their retention index and mass spectra using the Wiley 12 & NIST 2020 database.

### 3.6. Antioxidant Activity of Diatom Extracts

Prior to analyses, dried extracts were dissolved in 70% ethanol at a concentration of 10 mg/mL. Two assays, 2,2-diphenyl-1-picrylhydrazyl (DPPH) radical scavenging and oxygen radical absorbance capacity (ORAC) were used to determine the antioxidant potential of the extracts.

DPPH radical scavenging ability of diatom extracts was measured in 96-well microplates, following the method previously reported by Čagalj et al. [62]. Briefly, DPPH radical solution (290 µL) and prepared sample (10 µL) were pipetted in microplate wells. The absorbance was measured at 517 nm and the initial absorbance of the DPPH solution was set at 1.2. After 1 h, the decrease in the absorbance was measured using the plate reader (Synergy HTX Multi-Mode Reader, BioTek Instruments, Inc., Winooski, VT, USA). The antioxidant activity of extracts was expressed as DPPH radical inhibition percentages (% inhibition). ORAC method was performed using previously published protocols [63,64]. The samples were diluted in a 1:10 ratio and 25 µL of the prepared sample and 150 µL of fluorescein were added to each well of the microtiter plate. For 30 min, the plates were thermostated at 37 °C followed by the addition of 25 µL of AAPH. The measurements were recorded every minute over 80 min, at excitation and emission wavelengths of 485 and 520 nm. Results are expressed as µM Trolox equivalents (mM TE). Both assays were done in triplicate.

### 3.7. Statistical Analyses

Cell density data, collected and recorded using the Sedgewick Rafter method, were used for the analysis. The analyses of variance (one-way ANOVA followed by Fisher’s least significant difference test) was used to determine the significant changes (*p* < 0.05) in the growth rate in each system over time and the difference between the EIS and BRC systems, as well as antioxidant activities [65]. Analyses were performed using Statgraphics Centurion-Ver.16.1.11 (StatPoint Technologies, Inc., Warrenton, VA, USA). 

## 4. Conclusions

This study confirms that bioreactor cultivation is a successful system for the cultivation of diatoms. Optimal growth conditions in bioreactors allowed faster entry into the exponential and stationary growth phases. It could be hypothesized that prolongation of cultivation would result in slightly higher biomass in the bioreactor; however, the prolongation would also result in the death of diatom cells. The results of the chemical profiles revealed that oleamide was most abundant compound in the BRC and EIS systems, and that palmitelaidic acid, glycerol monostearate, myristic acid, cholesterol, eicosapentaenoic acid, 1-monopalmitin and 24-methylene cholesterol were also abundant. All of the mentioned compounds have been previously characterized for their biological activities, which is why some of them are already used in industry, while the other compounds have the potential for biotechnological applications. Moreover, in this study, tentative identification of derivatized 24-methylene cholesterol was obtained, which was not previously found in its derivatized form in the commercial libraries but is known from the literature. For precise identification techniques such HR-MS or NMR should be used. In addition, ethanolic extracts of *S. grevillei* showed low antioxidant activity at the tested concentration, so further studies should focus on the biological activity at higher concentrations and quantitative determination of the biologically active compounds. It should also be emphasized that, in addition to the extracts, silicate diatom frustules are also used in nanotechnology, which will certainly affect the profitability of the production of these microalgae in the future. Future research should focus on finding conditions under which it is possible to maximize the production of metabolites that have the potential for biotechnological applications.

## Figures and Tables

**Table 1:** List of tentatively identified compounds from *Skeletonema grevillei* extracts by GC-MS in bioreactor after 192 and 312 h.

No.	Retention Index		Similarty (%)		Proportion (%)		Identified Compound	Molar Weight
	BRC192	BRC312	BRC192	BRC312	BRC192	BRC312		
1	1242	n.d.	96	n.d.	0.05	n.d.	Benzoic acid, TMS derivative	194
2	1253	n.d.	86	n.d.	0.02	n.d.	Octanoic acid, TMS derivative	216
3	1269	1265	94	92	0.13	0.21	Glycerol, 3TMS derivative	308
4	1348	1345	95	92	0.08	0.05	Nonanoic acid, TMS derivative	230
5	1451	1450	95	91	0.02	0.02	Decanoic acid, TMS derivative	244
6	n.d.	1499	n.d.	94	n.d.	0.13	Malic acid, 3TMS derivative	350
7	1534	n.d.	91	n.d.	0.01	n.d.	1-Dodecanethiol	202
8	1653	1499	97	93	0.05	0.03	Dodecanoic acid, TMS derivative	272
9	1691	n.d.	85	n.d.	0.03	n.d.	Tetradecanenitrile	326
10	1753	1752	94	94	0.10	0.14	Tridecanoic acid, TMS derivative	286
11	1767	n.d.	91	n.d.	0.01	n.d.	1-Tetradecanol, TMS derivative	286
12	1788	1787	93	93	0.06	0.17	Loliolide, TMS	268
13	1853	1852	95	96	2.55	6.50	Myristic acid, TMS derivative	300
14	n.d.	1877	n.d.	90	n.d.	0.28	(*Z*)-3-Hexenyl β -glucopyranoside, 4TMS derivative	550
15	1879	n.d.	94	n.d.	0.11	n.d.	Palmitoleonitrile	235
16	1930	n.d.	86	n.d.	0.05	n.d.	(*E*)-13-Methyltetradec-9-enoic acid, TMS derivative	312
17	1952	1951	94	85	0.17	0.49	Pentadecanoic acid, TMS derivative	314
18	2009	2008	89	88	0.97	0.63	Eicosapentaenoic acid, TMS derivative	374
19	2031	2030	96	97	7.37	12.15	Palmitelaidic acid, TMS	326
20	2053	2052	96	95	2.31	2.73	Palmitic acid, TMS derivative	328
21	2076	n.d.	89	n.d.	0.43	n.d.	9-Octadecynenitrile	261
22	2083	2082	97	97	2.96	0.38	(*Z*)-9-Octadecenenitrile	263
23	2151	2094	92	83	0.04	0.03	Heptadecanoic acid, TMS derivative	342
24	n.d.	2157	n.d.	93	n.d.	0.04	Palmitoleamide	253
25	n.d.	2163	n.d.	87	n.d.	0.01	1-Octadecanol, TMS derivative	342
26	n.d.	2178	n.d.	85	n.d.	0.03	Octadecanamide	319
27	2184	2184	92	97	1.48	2.09	Phytol, TMS derivative	368
28	2216	2215	88	92	0.47	0.44	Linoleic acid, TMS derivate	352
29	2230	2229	94	94	2.36	0.90	(*Z*)-Oleic acid, TMS derivative	354
30	2251	2250	97	95	0.88	0.65	Stearic acid, TMS derivative	356
31	n.d.	2256	n.d.	86	n.d.	0.05	11-Methyloctadec-12-enoic acid, TMS derivative	368
32	n.d.	2331	n.d.	90	n.d.	0.16	(all-*Z*)-5,8,11,14,17-Eicosapentaenoic acid, methyl ester	330
33	2337	n.d.	92	n.d.	0.10	n.d.	(*Z*)-10-Nonadecenoic acid, TMS	368
34	2365	2362	95	96	2.30	2.50	9-Octadecenamide	281
35	n.d.	2373	n.d.	90	n.d.	0.68	(*Z*)-5,8,11-Eicosatrienoic acid, TMS derivative	378
36	2381	2380	94	96	3.29	6.00	Eicosapentaenoic acid, TMS derivative	374
37	2430	2422	83	82	49.84	33.65	Oleamide, TMS derivative	353
38	2450	2448	95	95	1.24	0.70	Steramide, TMS derivative	355
39	2564	2564	97	89	0.54	0.19	Doconexent, TMS derivative	400
40	2578	2578	93	87	1.39	1.09	2-Palmitoylglycerol, 2TMS derivative	474
41	2610	2610	96	96	2.91	2.89	1-Monopalmitin, 2TMS derivative	474
42	2771	2770	90	90	1.46	1.59	2-Monostearin, 2TMS derivative	502
43	2787	2787	93	90	0.34	0.10	1-Monooleoylglycerol, 2TMS derivative	500
44	2805	2804	97	96	5.92	9.30	Glycerol monostearate, 2TMS derivative	502
45	2826	2825	88	81	0.57	0.10	(*Z*)-Docos-13-enamide, TMS	409
46	n.d.	2997	n.d.	89	n.d.	0.15	Eicosanoic acid, 2,3-bis-(OTMS) propyl ester	530
47	2998	n.d.	93	n.d.	0.09	n.d.	2,3-Dihydroxypropyl icosanoate, 2TMS derivative	530
48	3066	3066	89	88	0.09	0.10	2-Arachidonoylglycerol, 2TMS derivative	522
49	3163	3162	96	96	3.97	6.21	Cholesterol, TMS derivative	458
50	n.d.	3201	n.d.	90	n.d.	0.40	Desmosterol, TMS derivative	456
51	3261	3260	90 *	88 *	3.03	5.09	24-Methylene cholesterol *	398 *
52	3380	3380	88	81	0.15	0.20	Isofucosterol, TMS	484
53	3672	3672	90	92	0.06	0.80	Oleanolic acid 2TMS	600

* A compound identified in non-derivatized form and its molecular weight (other data for this compound, RI, and proportion, were obtained for the derivatized form) as well as with literature data comparison for its derivatized form.; n.d.—not detected. BRC192—after 192 h in a bioreactor; BRC312—after 312 h in a bioreactor.

**Table 2:** List of tentatively identified compounds by GC-MS in incubation-shaking cabinet after 192 and 312 h.

No.	Retention Index		Similarity (%)		Proportion(%)		Identified Compound	Molar Weight
	EIS192	EIS312	EIS192	EIS312	EIS192	EIS312		
1	1335	n.d.	95	n.d.	0.10	n.d.	Nonanoic acid, TMS derivative	230
2	1444	1379	94	92	0.03	0.03	Decanoic acid, TMS derivative	244
3	n.d.	1448	n.d.	75	n.d.	0.01	Butanedioic acid, TMS derivate	350
4	1650	1630	93	95	0.04	0.05	Dodecanoic acid, TMS derivative	272
5	1691	n.d.	93	n.d.	0.02	n.d.	Tetradecanenitrile	209
6	1751	1739	88	93	0.03	0.13	Tridecanoic acid, TMS derivative	286
7	1787	1773	86	92	0.01	0.22	Loliolide, TMS	268
8	1805	1796	88	80	0.05	0.02	Azelaic acid, 2TMS derivative	332
9	n.d.	1831	n.d.	86	n.d.	0.06	Myristoleic acid, trimethylsilyl ester	298
10	1852	1845	95	96	1.59	5.12	Myristic acid, TMS derivative	300
11	1877	n.d.	94	n.d.	0.08	n.d.	Palmitoleonitrile	235
12	1898	n.d.	96	n.d.	0.19	n.d.	Heptadecanenitrile	251
13	n.d.	1956	n.d.	82	n.d.	0.03	Hexadecanenitrile	251
14	1951	1947	93	94	0.10	0.23	Pentadecanoic acid, TMS derivative	314
15	n.d.	1960	n.d.	93	n.d.	0.05	1-Hexadecanol, TMS derivative	314
16	1968	1964	96	95	0.66	0.25	Tetradecanamide	227
17	2031	2026	88	96	1.63	7.89	Palmitelaidic acid, TMS	326
18	2052	2049	96	96	1.65	2.07	Palmitic acid, TMS derivative	328
19	2076	n.d.	89	n.d.	0.43	n.d.	9-Octadecynenitrile	261
20	2082	2078	97	97	6.10	0.37	(*Z*)-9-Octadecenenitrile	263
21	2151	2149	91	80	0.04	0.02	Heptadecanoic acid, trimethylsilyl ester	342
22	2158	2155	97	96	0.97	0.36	Palmitoleamide	253
23	n.d.	2161	n.d.	86	n.d.	0.05	1-Octadecanol, TMS derivative	342
24	2178	2176	98	97	1.93	0.52	Hexadecanamide	255
25	2184	2183	93	97	0.20	2.06	Phytol, TMS derivative	368
26	n.d.	2213	n.d.	88	n.d.	0.24	Linoleic, TMS derivative	352
27	2251	2250	95	95	0.75	0.53	Stearic acid, TMS derivative	356
28	n.d.	2364	n.d.	96	n.d.	13,97	9-Octadecenamide	281
29	n.d.	2380	n.d.	96	n.d.	3.78	Eicosapentaenoic acid, TMS derivative	374
30	2427	2422	84	82	73.31	35,53	Oleamide, TMS derivative	353
31	2449	2419	95	95	1.37	0.75	Octadecanamide, N-TMS derivate	355
32	2571	n.d.	93	n.d.	0.81	n.d.	13-Docosenamide, (*Z*)-	337
33	2578	2577	93	94	0.32	1.88	2-Palmitoylglycerol, 2TMS derivative	474
34	2610	2610	94	96	0.47	6.92	1-Monopalmitin, 2TMS derivative	474
35	2771	2771	89	91	1.28	2.20	2-Monostearin, 2TMS derivative	502
36	2804	2804	95	96	3.52	10,64	Glycerol monostearate, 2TMS derivative	502
37	2998	2997	87	80	0.04	0.10	2,3-Dihydroxypropyl icosanoate, 2TMS derivative	530
38	3162	3160	96	96	1.21	1.97	Cholesterol, TMS derivative	458
39	3260	3529	88 *	89 *	1.05	1.59	24-Methylene cholesterol *	398 *
40	n.d.	3379	n.d.	88	n.d.	0.13	Isofucosterol, O-TMS	484
41	n.d.	3672	n.d.	90	n.d.	0.22	Oleanolic acid 2TMS	600

* A compound identified in non-derivatized form and its molecular weight (other data for this compound, RI, and proportion, were obtained for the derivatized form) as well as with literature data comparison for its derivatized form.; n.d.—not detected. EIS192—after 192 h in an incubation-shaking cabinet; EIS312—after 312 h in an incubation-shaking cabinet.

**Table 3:** The most dominant compounds identified from bioreactor and incubation-shaking cabinet in *Skeletonema grevillei* extracts.

No.	Compounds	Molecular formula	Structure	Properties
1.	Oleamide	C_18_H_35_NO	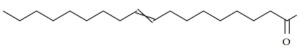	Anti-inflammatory, antialgal, antimicrobial and antifungal [30,31,34,35,36]
2.	Palmitelaidic acid	C_16_H_30_O_2_	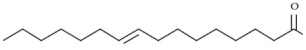	Antibiofilm activity [37]
3.	Glycerol monostearate	C_21_H_42_O_4_	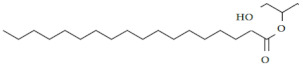	Anthelmintic [41]
4.	Myristic acid	C_14_H_28_O_2_	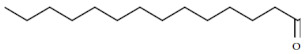	Antifungal, antiviral, anticancer and antiparasitic [40]
5.	Cholesterol	C_27_H_46_O	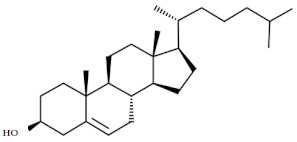	Anticancer, anticardiac, anti-inflammatory, antimicrobial, anti-psychotic, antioxidative [42]
6.	Eicosapentaenoic acid	C_20_H_30_O_2_	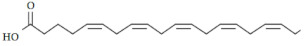	Cardioprotective, neuroprotective, anti-inflammatory, anticancer, antimicrobial and antioxidative [43,44,45,46]
7.	1-monopalmitin	C_19_H_38_O_4_	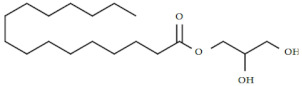	Antiviral [47]
8.	24-methylene cholesterol	C_28_H_46_O	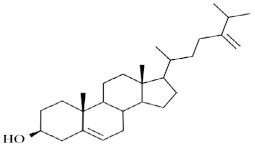	Anticancer [48]

## Data Availability

Data available on request.

## Supplementary Materials

The following supporting information can be downloaded at: https://www.mdpi.com/article/10.3390/md20110697/s1. Additional information of identified compound such as, retention time and m/z, available in Supplementary Table S1 List of identified compounds from *Skeletonema grevillei* extracts by GC-MS in incubation-shaking cabinet after 312 hours.
