# Supplementary material for: Comparison of Growth and Chemical Profile of Diatom Skeletonema grevillei in Bioreactor and Incubation-Shaking Cabinet in Two Growth Phases
Source: Mar Drugs. 2022 Nov 7;20(11):697. doi: 10.3390/md20110697 (PMC9695663; doi:10.3390/md20110697)
Supplement: Supplementary file 1 [file marinedrugs-20-00697-s001.zip › marinedrugs-2004973-supplementary.pdf]

Table S1. List of identified compounds from *Skeletonema grevillei* extracts by GC-MS in incubation-shaking cabinet after 192 hours.

No.	Retention time	Retention index	Identified compound	MS *m*/*z* (relative intensity)	Similarty (%)	Molar weight	Area	Proportion (%)
1	4.844	1335	Nonanoic acid, TMS derivative	216(15), 215(96), 145(15), 132(31), 131(21), 129(25), 117(84), 75(93), 73(100), 55(15)	95	230	565805	0,10
2	6.993	1444	Decanoic acid, TMS derivative	229(83), 145(14), 132(39), 131(22), 128(33), 117(90), 75(83), 73(100), 55(14), 41(17)	94	244	164887	0,03
3	8.317	1507	Hexanedioic acid, 2TMS derivative	275824), 172(21), 159(31), 146(36), 141(24), 117(29), 110(51), 74(67), 73(100), 54(29)	78	290	66251	0,01
4	11.442	1650	Dodecanoic acid, TMS derivative	257(92), 145(17), 132(55) 131(21), 129(40), 117(99), 74(82), 73(100), 55(19), 43(24)	93	272	201701	0,04
5	12.318	1691	Tetradecanenitrile	110(89), 97(100), 96(80), 83(57), 82(57), 70(49), 57(74), 55(51), 43(78), 41(72)	93	209	87904	0,02
6	13.587	1751	Tridecanoic acid, TMS derivative	272(23), 271(99), 145(21), 132(39), 129(31), 117(100), 75(60), 73(75), 54(19), 43(24)	88	286	186829	0,03
7	13.683	1758	Tetradecanamide	206(10), 75(16), 73(23), 72(38), 59(100), 57(10), 55(14), 44(16), 43(23), 41(19)	83	227	111560	0,02
8	14.347	1787	Loliolide, TMS	268(18), 253(35), 211(40), 178(54), 135(19), 133(19), 75(100), 73(61), 44(16), 43(34)	86	268	44086	0,01

9	14.722	1805	Azelaic acid, 2TMS derivative	317(34), 201(25), 148(24), 147(26), 131(46), 129(23), 116(23), 74(73), 73(100), 55(32)	88	332	305017	0,05
10	15.649	1852	Myristic acid, TMS derivative	286(17), 285(84), 145(24), 132(51), 129(40), 117(100), 75(68), 73(92), 55(20), 43(21)	95	300	9067143	1,59
11	16.178	1877	Palmitoleonitrile	136(61), 122(85), 83(32), 70(35), 69(72), 56(37), 55(100), 54(28), 43(39), 41(79)	94	235	429668	0,08
12	16.595	1898	Heptadecanenitrile	124(55), 110(83), 97(100), 96(77), 82(46), 70(49), 57(95), 55(61), 43(99), 41(83),	96	251	1106265	0,19
13	17.616	1951	Pentadecanoic acid, TMS derivative	300(20), 299(84), 145(29), 132(50), 129(38), 117(100), 74(61), 73(86), 55(18), 43(24)	93	314	599302	0,10
14	17.953	1968	Tetradecanamide	128(6), 86(6), 72(39), 60(7), 59(100), 57(6), 55(9), 44(9), 43(17), 41(13)	96	227	3666421	0,64
15	18.149	1978	(*Z*)-9-Octadecenenitrile	136(70), 122(65), 97(37), 83(38), 80(38), 69(80), 56(43), 55(100), 43(45), 41(83)	94	263	133518	0,02
16	19.097	2031	Palmitelaidic acid, TMS	311(75), 145(31), 129(61), 116(93), 96(27), 74(88), 73(100), 69(23), 55(49), 41(30)	88	326	9338939	1,63
17	19.505	2052	Palmitic Acid, TMS derivative	314(17), 313(69), 145(26), 132(51), 129(35), 117(100), 74(58), 73(81), 55(19), 43(23)	96	328	9455560	1,65
18	19.937	2076	9-Octadecynenitrile	96(35), 95(56), 82(47), 81(90), 79(33), 68(43), 67(100), 55(62), 54(41), 41(60)	89	261	2434268	0,43

19	20.072	2082	(*Z*)-9-Octadecenenitrile	136(69), 122(85), 97(42), 83(52), 70(40), 69(74), 56(41), 55(100), 43(49), 41(82)	97	263	34869389	6,10
20	21.311	2151	Heptadecanoic acid, trimethylsilyl ester	328(20), 327(78), 145(29), 132(57), 128(40), 117(100), 75(63), 73(94), 43(28), 41(16)	91	342	235014	0,04
21	21.432	2158	Palmitoleamide	126(14), 98(11), 72(69), 69(17), 60(10), 59(100), 55(31), 44(15), 43(21), 41(27)	97	253	5559867	0,97
22	21.814	2178	Hexadecanamide	128(7), 86(6), 72(43), 60(8), 59(100), 57(8), 55(10), 44(8), 43(19), 41(12)	98	255	11036086	1,93
23	21.908	2184	Phytol, TMS derivative	144(14), 143(100), 123(15), 81(9), 74(19), 73(24), 59(7), 57(7), 55(7), 43(11)	93	368	1129242	0,20
24	23.042	2251	Stearic acid, TMS derivative	341(22), 145(23), 132(53), 129(36), 117(100),75(35), 73(62), 55(15), 43(17), 41(9)	95	356	4280221	0,75
25	25.955	2423	Oleamide, TMS derivative	353(24), 338(56), 144(74), 131(100), 128(42), 116(50), 75(61), 73(58), 55(22), 41(19)	84	353	161851002	28,29
26	26.028	2431	Oleamide, TMS derivative	353(20), 338(39), 144(46), 131(100), 128(33), 116(43), 75(59), 73(54), 55(24), 41(19)	82	353	257549477	45,02
27	26.303	2449	Octadecanamide, N-TMS derivate	341(22), 341(5), 144(13), 132(11), 131(100), 128(7), 116(27), 75(21), 73(24), 43(10),	95	355	7824268	1,37
28	28.188	2571	(*Z*)-13-Docosenamide	126(16), 83(16), 72(78), 69(25), 60(15), 59(100), 57(19), 55(47), 43(32), 41(32)	93	337	4649338	0,81

29	28.308	2578	2-Palmitoylglycerol, 2TMS derivative	313(27), 217(93), 202(29), 147(48), 129(100), 103(37), 73(71), 57(23), 55(22), 43(29)	93	474	1822253	0,32
30	28.787	2610	1-Monopalmitin, 2TMS derivative	372(29), 371(100), 239(14), 202(12), 147(24), 129(13)73(28), 71(10), 57(16), 43(15)	94	474	2677655	0,47
31	31.104	2771	2-Monostearin, 2TMS derivative	341(23), 218(85), 203(26), 191(22), 147(42), 129(100), 103(38), 73(56), 57(22), 43(26)	89	502	7314710	1,28
32	31.583	2804	Glycerol monostearate, 2TMS derivative	400(24), 399(100), 203(13), 147(20), 129(15), 73(29), 71(10), 57(16), 55(9) ,43(15)	95	502	20125448	3,52
33	34.199	2998	2,3-Dihydroxypropyl icosanoate, 2TMS derivative	429(12), 428(32), 427(100), 203(17), 147(25), 129(20), 73(33), 71(11), 57(20), 43(17)	87	530	239422	0,04
34	36.261	3162	Cholesterol, TMS derivative	458(48), 368(81), 353(37), 329(78), 129(100), 121(34), 95(39), 81(32), 75(33), 73(49)	96	458	6916629	1,21
35	37.502	3260	24-Methylene cholesterol	386(47), 129(100), 119(42), 107(33), 95(40), 81(37), 74(36), 73(60), 69(43), 55(41)			6010162	1,05

Table S2. List of identified compounds from *Skeletonema grevillei* extracts by GC-MS in incubation-shaking cabinet after 312 hours.

No.	Retention time (min)	Retention index	Identified compound	MS *m/z* (relative intensity)	Similarty (%)	Molar weight	Area	Proportion (%)
1	5.667	1379	Decanoic acid, TMS derivative	335(8), 244(14), 234(8), 232(21),189(10), 148(8), 147(48), 74(11), 73(100), 45(8)	92	244	160102	0,04
2	7.061	1448	Butanedioic acid, TMS derivate	244(12), 232(23), 189(16), 147(64), 133(12), 75(10), 73(100), 55(16), 45(13), 43(10)	75	350	48587	0,01
3	11.004	1630	Dodecanoic acid, TMS derivate	258(16), 132(43), 131(15), 129(35), 117(100), 74(74), 73(92), 55(19), 43(15), 41(17)	95	272	206258	0,05
4	13.336	1739	Tridecanoic acid, TMS derivative	271(80), 144(21), 132(38),129(34), 117(95), 74(76), 73(100),55(21), 43(21), 41(19)	93	286	591014	0,13
5	14.053	1773	Loliolide, TMS	268(18), 252(59), 211(41), 178(56), 162(17), 135(17), 133(22), 74(100), 73(56), 43(41)	92	268	996732	0,22
6	14.526	1796	Azelaic acid, 2TMS ester	317(30), 201(37), 152(23), 148(25), 147(25), 131(48), 116(31), 74(66), 73(100), 55(41)	80	332	96955	0,02
7	15.258	1831	Myristoleic acid, trimethylsilyl ester	283(79), 145(24), 131(31), 129(60), 116(78), 96(26), 74(94), 73(100), 55(44), 41(20)	86	298	267138	0,06
8	15.512	1845	Myristic acid, TMS derivative	285(72), 145(23), 132(44), 129(34), 117(100), 75(64), 73(87), 55(19), 43(20), 41(15)	96	300	23200617	5,12

9	16.468	1956	Hexadecanenitrile	110(66), 97(76), 96(61), 83(46), 75(53), 73(99), 57(90), 55(67), 43(100), 41(87)	82	251	152460	0,03
10	17.533	1947	Pentadecanoic acid, TMS derivative	300(21), 299(81), 145(24), 132(49), 129(38), 117(100), 75(56), 73(83), 55(17), 43(22)	94	314	1051806	0,23
11	17.825	1960	1-Hexadecanol, TMS derivative	300(30), 299(100), 103(11), 97(15), 88(6), 83(10), 75(22), 71(6), 57(8), 43(8)	93	314	238211	0,05
12	17.868	1964	Tetradecanamide	86(7), 73(7), 72(38), 60(7), 59(100), 57(5), 55(9), 44(9), 43(19), 41(13)	95	227	1137392	0,25
13	19.051	2026	Palmitelaidic acid, TMS	486(1), 479(1), 395(1), 320(1), 317(2), 307(2), 288(2), 287(17), 233(1), 59(100)	96	326	35754904	7,89
14	19.458	2049	Palmitic Acid, TMS derivative	313(28), 145(27), 132(55), 129(37), 117(100), 74(66), 73(90), 55(24), 43(29), 41(18)	96	328	9373599	2,07
15	19.995	2078	(*Z*)-9-Octadecenenitrile	136(63), 122(85), 97(43), 83(50), 70(37), 69(69), 56(39), 55(100), 43(44), 41(75)	97	263	1670032	0,37
16	21.284	2149	Heptadecanoic acid, trimethylsilyl ester	327(82), 145(31), 132(53), 129(45), 116(100), 74(57), 73(55), 59(43), 55(30), 41(26)	80	342	84649	0,02
17	21.390	2155	Palmitoleamide	126(14), 112(11), 72(72), 69(16), 60(11), 59(100), 55(34), 44(16), 43(22), 41(28)	96	253	1650580	0,36
18	21.492	2161	1-Octadecanol, TMS derivative	328(29), 327(100), 97(9), 75(22), 73(13), 72(11), 69(9), 59(18), 55(9), 43(12)	86	342	235080	0,05

19	21.768	2176	Hexadecanamide	128(7), 86(6), 72(44), 60(9), 59(100), 57(8), 55(11), 44(8), 43(19), 41(13)	97	255	2374640	0,52
20	21.882	2183	Phytol, TMS derivative	144(12), 143(100), 124(5), 123(14), 81(8), 75(18), 73(24), 57(7), 55(5), 43(8)	97	368	9333113	2,06
21	22.420	2213	Linoleic, TMS derivative	337(42), 144(37), 131(67), 116(48), 81(38), 74(100), 73(96), 67(45), 55(40), 41(32)	88	352	1084296	0,24
22	23.017	2250	Stearic acid, TMS derivative	342(17), 341(61), 145(31), 132(53), 129(38), 117(100), 74(39), 73(60), 55(17), 43(20)	95	356	2380761	0,53
23	24.950	2364	9-Octadecenamide	126(16), 112(12), 72(72), 69(16), 60(12), 59(100), 55(33), 44(13), 43(23), 41(26)	96	281	63315094	13,97
24	25.210	2380	Eicosapentaenoic Acid, TMS derivative	119(46), 117(63), 106(42), 105(46), 93(49), 91(75), 79(100), 73(89), 74(75), 67(54)	96	374	17129637	3,78
25	25.889	2422	Oleamide, TMS derivative	353(22), 338(55), 198(19), 144(65), 131(100), 128(41), 116(49), 74(61), 73(55), 55(22)	82	353	161013588	35,53
26	26.274	2419	Octadecanamide, N-TMS derivate	341(6), 340(21), 144(12), 132(11), 131(100), 128(7), 116(30), 74(23), 73(25), 43(11)	95	355	3410043	0,75
27	28.296	2577	2-Palmitoylglycerol, 2TMS derivative	313(28), 218(90), 202(28), 190(22), 147(44), 129(100), 103(41), 73(69), 57(22), 43(26)	94	474	8519649	1,88
28	28.786	2610	1-Monopalmitin, 2TMS derivative	459(9), 372(30), 371(100), 239(14), 203(12), 147(20), 129(13), 73(26), 57(14), 43(12)	96	474	31376697	6,92

29	31.101	2771	2-Monostearin, 2TMS derivative	341(23), 218(21), 217(94), 202(28), 147(43), 129(100), 103(39), 73(54), 57(21), 43(24)	91	502	9975855	2,20
30	31.589	2804	Glycerol monostearate, 2TMS derivative	487(10), 400(30), 399(100), 202(14), 147(21), 129(16), 73(28), 71(9), 57(16), 43(15)	96	502	48211119	10,64
31	34.183	2997	2,3-Dihydroxypropyl icosanoate, 2TMS derivative	515(13), 428(27), 427(100), 203(14), 147(15), 129(14), 73(30), 71(10), 57(17), 43(15)	80	530	431474	0,10
32	36.249	3160	Cholesterol, TMS derivative	458(40), 368(66), 353(34), 329(74), 129(100), 121(30), 95(37), 81(32), 74(32), 73(48)	96	458	8931990	1,97
33	37.490	3529	24-Methylene cholesterol	386(39), 129(100), 119(43), 107(37), 95(42), 81(43), 74(38), 73(62), 69(46), 55(49)			7198459	1,59
34	39.017	3379	Isofucosterol, O-TMS	387(37), 386(100), 296(96), 280(55), 129(59), 95(37), 81(33), 73(64), 69(34), 55(57)	88	484	579016	0,13
35	42.703	3672	Oleanolic acid 2TMS	482(32), 320(31), 204(15), 203(100), 202(56), 190(27), 189(32), 133(16), 129(14), 73(45)	90	600	979409	0,22

Table S3. List of identified compounds from *Skeletonema grevillei* extracts by GC-MS in bioreactor after 192 hours.

No.	Retention time (min)	Retention index	Identified compound	MS *m/z* (relative intensity)	Similarty (%)	Molar weight	Area	Proportion (%)
1	3.099	1242	Benzoic Acid, TMS derivative	193(11), 179(14), 178(100), 136(9), 135(69), 106(6), 105(75)	96	194	555380	0,05
2	3.298	1253	Octanoic acid, TMS derivative	201(74), 132(18), 131(18), 129(17), 117(61), 75(91), 73(100), 70(16), 55(28), 41(23)	86	216	196879	0,02
3	3.599	1269	Glycerol, 3TMS derivative	218(21), 206(11), 205(60), 148(11), 147(80), 133(16), 117(35), 103(32), 73(100), 45(9)	94	308	1610692	0,13
4	5.082	1348	Nonanoic acid, TMS derivative	216(14), 215(90), 145(13), 132(32), 131(22), 129(27), 117(80), 75(91), 73(100), 55(15)	95	230	971265	0,08
5	7.144	1451	Decanoic acid, TMS derivative	229(73), 132(28), 131(19), 129(25), 117(76), 75(96), 73(100), 55(17), 43(18), 41(16),	95	244	224588	0,02
6	8.909	1534	1-Dodecanethiol	97(71), 87(56), 83(75), 70(74), 69(87), 57(65), 56(78), 55(99), 43(100), 41(87)	91	202	166837	0,01

7	11.499	1653	Dodecanoic acid, TMS derivative	258(18), 257(86), 145(19), 132(39), 131(18), 129(34), 117(100), 75(69), 73(86), 55(19)	97	272	589219	0,05
8	12.319	1691	Tetradecanenitrile	110(73), 97(90), 96(64), 83(53), 82(53), 69(45), 57(61), 55(100), 43(70), 41(72)	85	326	395427	0,03
9	13.621	1753	Tridecanoic acid, TMS derivative	272(17), 271(88), 145(21), 132(47), 131(17), 129(39), 117(100), 74(70), 73(91), 55(19)	94	286	1154450	0,10
10	13.918	1767	1-Tetradecanol, TMS derivative	272(25), 271(100), 102(15), 97(14), 83(14), 74(36), 73(18), 69(9), 57(8), 43(9)	91	286	145545	0,01
11	14.350	1788	Loliolide, TMS	268(20), 253(63), 225(20), 211(47), 178(63), 163(18), 133(21), 78(63), 75(100), 73(57), 42(36)	93	268	713285	0,06
12	14.724	1805	Azelaic acid, 2TMS derivative	317(65), 201(39), 152(24), 148(36), 146(22), 128(31), 117(21), 74(68), 73(100), 55(41)	82	332	76487	0,01
13	15.678	1853	Myristic acid, TMS derivative	286(19), 285(83), 145(23), 132(49), 129(38), 117(100),75(33), 73(85), 55(19), 43(20)	95	300	30410779	2,55

14	16.198	1879	Palmitoleonitrile	136(66), 122(82), 83(37), 70(37), 69(71), 56(38), 55(100), 54(30), 43(34), 41(83)	94	235	1284500	0,11
15	17.158	1930	(*E*)-13-Methyltetradec-9-enoic acid, TMS derivative	311(43), 145(33), 129(82), 117(96), 96(25), 75(94), 73(100), 55(62), 43(23), 41(30)	86	312	612221	0,05
16	17.633	1952	Pentadecanoic acid, TMS derivative	300(20), 299(85), 145(23), 132(49), 129(37), 117(100), 75(57), 73(81), 55(17), 43(21)	94	314	2055261	0,17
17	18.625	2009	Eicosapentaenoic acid, TMS derivative	117)39), 107(34), 105(40), 93(36), 91(61), 80(46), 79(93), 74(89), 73(100), 67(38)	89	374	11598665	0,97
18	19.138	2031	Palmitelaidic acid, TMS	311(52), 145(31), 129(69), 117(86), 96(28), 81(24), 74(95), 73(100), 55(54), 41(33)	96	326	87911301	7,37
19	19.525	2053	Palmitic Acid, TMS derivative	314(16), 313(75), 145(27), 132(52), 129(38), 117(100), 74(55), 73(78), 55(19), 43(23)	96	328	27511060	2,31
20	19.952	2076	9-Octadecynenitrile	96(32), 95(52), 82(47), 81(92), 79(32), 68(41), 67(100), 55(55), 54(41), 41(56)	89	261	5105299	0,43

21	20.083	2083	(*Z*)-9-Octadecenenitrile	136(56), 122(77), 97(38), 83(49), 70(37), 69(69), 56(39), 55(100), 43(46), 41(79)	97	263	35357695	2,96
22	21.313	2151	Heptadecanoic acid, TMS derivative	328(19), 327(89), 145(33), 132(50), 129(35), 117(100), 74(55), 73(80), 55(21), 43(22)	92	342	515709	0,04
23	21.913	2184	Phytol, TMS derivative	203(6), 144(12), 143(100), 123(15), 81(8), 74(19), 73(28), 27(7), 55(5), 43(9)	92	368	17636940	1,48
24	22.392	2216	Linoleic, TMS derivate	337(50), 131(62), 116(48), 95(32), 81(50), 75(100), 73(91), 67(57), 55(41), 41(34)	88	352	5597272	0,47
25	22.703	2230	(*Z*)-Oleic Acid, TMS derivative	339(67), 145(37), 129(63), 116(85), 96(30), 74(90), 73(100), 69(30), 55(55), 41(31)	94	354	28106811	2,36
26	23.033	2251	Stearic acid, TMS derivative	342(25), 341(98), 145(29), 132(51), 129(35), 117(100), 75(37), 73(59), 55(16), 43(20)	97	356	10492836	0,88
27	24.494	2337	(*Z*)-10-Nonadecenoic acid,TMS	353(52), 145(34)129(53), 116(77), 96(28), 74(84), 73(100), 69(32), 55(55), 41(29)	92	368	1228482	0,10

28	24.969	2365	9-Octadecenamide	126(15), 112(13), 72(70), 69(16), 60(12), 59(100), 55(32), 44(12), 43(22), 41(25)	95	281	27499610	2,30
29	25.235	2381	Eicosapentaenoic Acid, TMS derivative	119(44), 117(60), 106(40), 105(43), 93(48), 91(76), 79(100), 74(75), 73(89), 67(54)	94	374	39206904	3,29
30	26.024	2430	Oleamide, TMS derivative	353(23), 338(57), 198(22), 144(80), 131(100), 128(43), 116(49), 74(57), 73(58), 55(22)	83	353	594746694	49,84
31	26.267	2450	Steramide, TMS derivative	341(6), 340(21), 144(14), 32(12), 131(100), 128(8), 116(28), 74(22), 73(26), 43(10)	95	355	14833820	1,24
32	28.037	2560	2-Palmitoleoylglycerol bis-trimethylsilyl ether	218(33), 147(40), 129(96), 117(19), 103(100), 75(24), 73(92), 67(18), 55(30), 41(18)	90	472	5932037	0,50
33	28.082	2564	Doconexent, TMS derivative	119(37), 117(55), 108(38), 105(34), 93(42), 91(67), 79(86), 75(45), 73(100), 67(46)	97	400	6477284	0,54
34	28.307	2578	2-Palmitoylglycerol, 2TMS derivative	313(23), 217(84), 203(26), 190(21), 147(48), 129(100), 103(37),73(62), 57(21), 43(24)	93	474	10599223	0,89

35	28.798	2610	1-Monopalmitin, 2TMS derivative	372(25), 371(100), 239(14), 203(11), 147(22), 129(11), 73(26), 71(9), 57(15), 43(14)	96	474	34669685	2,91
36	31.105	2771	2-Monostearin, 2TMS derivative	341(30), 218(91), 203(27), 191(21), 147(42), 129(100), 103(39), 73(54), 57(22), 43(25)	90	502	17380065	1,46
37	31.341	2787	1-Monooleoylglycerol, 2TMS derivative	397(80), 203(27), 201(19), 147(54), 129(100), 103(41), 83(20), 73(80), 69(31), 55(41)	93	500	4083277	0,34
38	31.598	2805	Glycerol monostearate, 2TMS derivative	400(30), 399(100), 202(15), 147(24), 129(18), 73(34), 71(12), 57(20), 55(11), 43(19)	97	502	70689560	5,92
39	31.871	2826	(*Z*)-Docos-13-enamide, TMS	409(30), 394(38), 144(52), 131(100), 128(27), 116(34), 75(49), 73(46), 55(24), 43(16)	88	409	6825007	0,57
40	34.110	2998	2,3-Dihydroxypropyl icosanoate, 2TMS derivative	428(35), 427(100), 206(21), 203(19), 147(28), 129(28), 75(19), 73(61), 57(24), 43(26)	93	530	1066093	0,09
41	35.056	3066	2-Arachidonoylglycerol, 2TMS derivative	147(19), 131(13), 129(45), 108(15), 105(14), 103(100), 91(20), 79(24), 73(67), 67(18)	89	522	1053023	0,09

42	36.278	3163	Cholesterol, TMS derivative	458(46), 368(76), 353(36), 329(87), 129(100), 121(32), 95(38), 81(30), 74(32), 73(47)	96	458	47323395	3,97
43	37.511	3261	24-Methylene cholesterol	386(50), 296(40), 129(100), 119(41), 107(34), 95(41), 81(38), 73(55), 69(41), 55(40)			36156623	3,03
44	39.013	3380	Isofucosterol, TMS	387(32), 386(100), 296(84), 281(42), 129(67), 119(33), 74(28), 73(47), 69(38), 55(51)	88	484	1804338	0,15
45	42.703	3672	Oleanolic acid 2TMS	472(53), 367(25), 280(37), 207(21), 206(100), 129(36), 74(32), 73(85), 55(24), 43(26)	90	600	766031	0,06

Table S4. List of identified compounds from *Skeletonema grevillei* extracts by GC-MS in bioreactor after 312 hours.

No.	Retention time (min)	Retention index	Identified compound	MS *m/z* (relative intensity)	Similarty (%)	Molar weight	Area	Proportion (%)
1	3.527	1265	Glycerol, 3TMS derivative	298(14), 217(20), 205(10), 204(55), 148(11), 147(72), 133(16), 116(32), 103(31), 73(100)	92	308	979306	0,21
2	5.027	1345	Nonanoic acid, TMS derivative	215(84), 132(29), 131(22), 129(22), 117(86), 74(94), 73(100), 55(16), 43(15), 41(13)	92	230	227297	0,05
3	7.115	1450	Decanoic acid, TMS derivative	230(15), 229(100), 144(15), 132(34), 131(20), 128(28), 117(86), 74(82), 73(72), 55(20)	91	244	101778	0,02
4	8.143	1499	Malic acid, 3TMS derivative	244(11), 232(23), 189(9), 148(9), 147(54),133(10), 75(8), 74(8), 73(100), 54(8)	94	350	635539	0,13
5	11.485	1499	Dodecanoic acid, trimethylsilyl ester	258(20), 257(85), 132(39), 131(20), 129(37), 116(100), 74(70), 73(93), 55(18), 43(17)	93	272	130045	0,03
6	13.611	1752	Tridecanoic acid, TMS derivative	272(19), 271(90), 145(18), 132(44), 129(35), 116(100), 74(67), 73(85), 55(19), 43(18)	94	286	651857	0,14

7	14.339	1787	Loliolide, TMS	268(19), 252(66), 211(42), 178(57), 163(19), 135(20), 133(20), 74(100), 73(58), 43(37)	93	268	788245	0,17
8	15.673	1852	Myristic acid, TMS derivative	286(16), 285(75), 145(22), 132(46), 129(36), 116(100),74(62), 73(84), 55(19), 43(19)	96	300	30912180	6,50
9	16.179	1877	(*Z*)-3-Hexenyl-β-glucopyranoside, 4TMS derivative	218(15), 217(50), 216(100), 204(18), 190(20), 147(21), 132(15), 129(44), 116(14), 73(80)	90	550	1312557	0,28
10	17.628	1951	Pentadecanoic acid, TMS derivative	299(59), 216(26), 145(22), 132(42), 129(34), 116(90), 74(51), 73(100), 55(15), 43(17)	85	314	2332135	0,49
11	18.722	2008	Eicosapentaenoic Acid, TMS derivative	117(30), 107(37), 105(43), 93(32), 91(62), 80(45), 79(100), 74(83), 73(80), 67(33),	88	374	2998226	0,63
12	19.124	2030	Palmitelaidic acid, TMS	311(32), 145(27), 129(58), 116(85), 96(26), 7(91), 73(100), 55(50), 43(23), 41(33),	97	326	57775151	12,15
13	19.516	2052	Palmitic Acid, TMS derivative	313(68), 314(18), 145(27), 132(51), 129(36), 116(100), 74(57), 73(79), 55(19), 43(23)	95	328	12974547	2,73

14	20.060	2082	(*Z*)-9-Octadecenenitrile	136(60), 122(77), 97(37), 83(50), 70(40), 69(73), 56(38), 55(100), 43(46), 41(79)	97	263	1802523	0,38
15	20.285	2094	Heptadecanoic acid, TMS derivative	327(89), 145(38), 132(52), 129(57), 117(100), 74(75), 73(70), 57(58), 55(36), 43(62)	83	342	157516	0,03
16	21.429	2157	Palmitoleamide	126(14), 112(11), 72(73), 69(15), 60(10), 59(100), 55(35), 44(13), 43(23), 41(30),	93	253	175511	0,04
17	21.531	2163	1-Octadecanol, TMS derivative	328(24), 327(100), 103(16), 97(17), 83(16), 75(33), 73(22), 57(13), 55(12), 43(12)	87	342	69796	0,01
18	21.801	2178	Octadecanamide	128(6), 86(5) 73(6), 72(44), 60(7), 59(100), 57(7), 44(9), 43(15), 41(8)	85	319	131621	0,03
19	21.910	2184	Phytol, TMS derivative	144(11), 143(100), 124(5), 123(14), 81(9), 75(18), 73(24), 57(6), 55(5), 43(8)	97	368	9943488	2,09
20	22.456	2215	(*Z*, *Z*)-9,12-Octadecadienoic acid, TMS derivative	337(57), 262(37), 129(37), 95(50), 82(36), 81(69), 74(94), 73(100), 67(71), 55(48)	92	352	2080686	0,44
21	22.689	2229	(*Z*)-Oleic Acid, TMS derivative	339(58), 145(27), 129(57), 117(62), 96(23), 74(81), 73(100), 69(26), 55(46), 41(28)	94	354	4278517	0,90

22	23.036	2250	Stearic acid, TMS derivative	342(21), 341(78), 145(34), 132(57), 129(41), 117(100), 74(60), 73(86), 55(23), 43(30)	95	356	3070858	0,65
23	23.133	2256	11-Methyloctadec-12-enoic acid, TMS derivative	353(100), 229(64), 129(37), 117(56), 83(37), 75(70), 73(72), 69(78), 55(90), 41(30)	86	368	249651	0,05
24	24.396	2331	(all-*Z*)-5,8,11,14,17-Eicosapentaenoic acid, methyl ester	119(39), 106(33), 105(40), 93(48), 91(73), 80(34), 79(100), 77(30), 67(53), 41(37)	90	330	748759	0,16
25	24.912	2362	9-Octadecenamide, (*Z*)-	72(71), 69(11), 67(13), 60(9), 59(100), 57(14), 55(35), 44(16), 43(27), 41(38)	96	281	11871750	2,50
26	25.095	2373	(*Z*)-5,8,11-Eicosatrienoic acid, TMS derivative	117(59), 106(45), 105(34), 93(55), 91(58), 80(67), 79(88), 75(82), 73(100), 67(59)	90	378	3226506	0,68
27	25.224	2380	Eicosapentaenoic Acid, TMS derivative	119(42), 117(63), 106(43), 105(47), 93(52), 91(79), 79(100), 74(80), 73(95), 67(57)	96	374	28541554	6,00
28	25.896	2422	Oleamide, TMS derivative	338(53), 353(26), 144(79), 131(100), 128(39), 116(52), 74(65), 73(61), 55(22), 41(19)	82	353	137888236	29,00

29	25.985	2428	Oleamide, TMS derivative	353(23), 338(33), 144(54), 131(100), 128(32), 116(40), 74(55), 73(51), 55(22), 43(17)	82	353	20828960	4,38
30	26.158	2438	Oleamide, TMS derivative	338(40), 353(19), 144(48), 131(100), 128(36), 116(43), 74(66), 73(53), 55(34), 41(24)	79	353	1298335	0,27
31	26.280	2448	N-Trimethylsilylstearamide	340(21), 144(13), 132(13), 131(100), 128(7), 116(31), 75(23), 73(29), 43(12), 41(6)	95	355	3309798	0,70
32	28.075	2564	Doconexent, TMS derivative	119(45), 117(63), 108(38), 105(43), 93(50), 91(84), 79(99), 74(57), 73 (100), 67(54)	89	400	903754	0,19
33	28.299	2578	2-Palmitoylglycerol, 2TMS derivative	313(21), 218(73), 203(25), 190(20), 147(54), 129(100), 103(43), 73(84), 55(21), 43(21)	87	474	5176829	1,09
34	28.782	2610	1-Monopalmitin, 2TMS derivative	372(37), 371(100), 239(19), 204(12), 202(16), 147(29), 129(16), 73(35), 57(19), 43(17)	96	474	13751047	2,89
35	31.099	2770	2-Monostearin, 2TMS derivative	341(28), 217(95), 202(25), 190(22), 147(43), 129(100), 103(37), 73(55), 57(22), 43(24)	90	502	7539368	1,59

36	31.332	2787	1-Monooleoylglycerol, 2TMS derivative	398(25), 397(57), 204(24), 202(28), 147(49), 129(100), 102(47), 95(26), 73(88), 55(30)	90	500	455591	0,10
37	31.584	2804	Glycerol monostearate, 2TMS derivative	400(28), 399(100), 202(14), 147(22), 129(17), 73(30), 71(10), 57(17), 55(10), 43(16)	96	502	44230013	9,30
38	31.862	2825	(*Z*)-Docos-13-enamide, N-TMS	409(27), 394(34), 144(50), 131(100), 128(28), 116(30), 74(51), 73(62), 55(21), 43(14)	81	409	453533	0,10
39	34.181	2997	Eicosanoic acid, 2,3-bis-(OTMS)propyl ester	428(33), 427(100), 205(10), 202(14), 147(21), 129(19), 103(10), 73(38), 57(15), 43(13)	89	530	718301	0,15
40	35.049	3066	2-Arachidonoylglycerol, 2TMS derivative	147(18), 129(45), 108(12), 105(15), 103(100), 93(14), 91(19), 79(25), 73(66), 67(14)	88	522	460175	0,10
41	36.260	3162	Cholesterol, TMS derivative	458(49), 368(92), 353(36), 329(87), 129(100), 121(31), 95(39), 81(31), 74(33), 73(48)	96	458	29524236	6,21
42	36.749	3201	Desmosterol, TMS derivative	343(78), 253(53), 133(51), 129(100), 119(77), 95(43), 81(39), 75(41), 73(90), 69(90)	90	456	1900720	0,40

43	37.499	3260	24-Methylene cholesterol	386(51), 296(36), 129(100), 119(43), 95(41), 81(41), 74(37), 73(61), 69(44), 55(42)			24216310	5,09
44	39.010	3380	Isofucosterol, O-TMS	387(35), 386(100), 296(68), 281(32), 129(69), 119(32), 95(31), 73(47), 69(39), 55(54)	81	484	929026	0,20
45	42.713	3672	Oleanolic acid 2TMS	473(39), 472(100), 471(39), 367(95), 159(35), 135(42), 133(51), 129(82), 73(82), 43(28)	92	600	3804734	0,80
